# Evaluation of HLA-G Expression in Multipotent Mesenchymal Stromal Cells Derived from Vitrified Wharton’s Jelly Tissue

**DOI:** 10.3390/bioengineering5040095

**Published:** 2018-11-01

**Authors:** Panagiotis Mallis, Dimitra Boulari, Efstathios Michalopoulos, Amalia Dinou, Maria Spyropoulou-Vlachou, Catherine Stavropoulos-Giokas

**Affiliations:** 1 Hellenic Cord Blood Bank, Biomedical Research Foundation Academy of Athens, 4 Soranou Ephessiou Street, 115 27 Athens, Greece; pmallis@bioacademy.gr (P.M.); dimitra.mpoulari@gmail.com (D.B.); adinou@bioacademy.gr (A.D.); cstavrop@bioacademy.gr (C.S.-G.); 2 Immunology Department-Tissue Typing Lab, “Alexandra” General Hospital of Athens, Lourou Street, 11528 Athens, Greece; marilynspy@yahoo.gr

**Keywords:** Mesenchymal Stromal Cells, Wharton’s Jelly tissue, HLA-G, mixed lymphocyte reaction, vitrification, VS55, long term storage

## Abstract

Background: Mesenchymal Stromal Cells (MSCs) from Wharton’s Jelly (WJ) tissue express HLA-G, a molecule which exerts several immunological properties. This study aimed at the evaluation of HLA-G expression in MSCs derived from vitrified WJ tissue. Methods: WJ tissue samples were isolated from human umbilical cords, vitrified with the use of VS55 solution and stored for 1 year at −196 °C. After 1 year of storage, the WJ tissue was thawed and MSCs were isolated. Then, MSCs were expanded until reaching passage 8, followed by estimation of cell number, cell doubling time (CDT), population doubling (PD) and cell viability. In addition, multilineage differentiation, Colony-Forming Units (CFUs) assay and immunophenotypic analyses were performed. HLA-G expression in MSCs derived from vitrified samples was evaluated by immunohistochemistry, RT-PCR/PCR, mixed lymphocyte reaction (MLR) and immunofluorescence. MSCs derived from non-vitrified WJ tissue were used in order to validate the results obtained from the above methods. Results: MSCs were successfully obtained from vitrified WJ tissues retaining their morphological and multilineage differentiation properties. Furthermore, MSCs from vitrified WJ tissues successfully expressed HLA-G. Conclusion: The above results indicated the successful expression of HLA-G by MSCs from vitrified WJ tissues, thus making them ideal candidates for immunomodulation.

## 1. Introduction

Multipotent Mesenchymal Stromal Cells, also known as Mesenchymal Stem Cells can efficiently be used in a wide variety of tissue engineering and regenerative medicine approaches, such as treatment of bone disorders and regeneration of cardiovascular tissue [1,2,3]. In addition, MSCs are characterized by critical immunomodulatory properties and could be ideal candidates for the regulation of the immune response [4,5].

According to the International Society for Cellular Therapies (ISCT), MSCs are a fibroblastic cell population, which can be differentiated under defined conditions to mesodermal lineages such as “adipocytes”, “osteocytes” and “chondrocytes” [6]. Moreover, human MSCs express specific clusters of differentiation (CDs), including CD73 (ecto-5’-nucleotidase), CD105 (endoglin), CD90 (Thy-1), while lacking totally the expression of CD45 (lymphocyte common antigen), CD34 (hematopoietic stem cell antigen) and HLA class II [6].

MSCs are well known for their immunomodulatory-immunosuppressive properties and their potential use in graft-versus-host disease (GVHD) and autoimmune disorders [7,8,9].

The immunomodulation, which is induced by MSCs, can be performed either with cell-cell interaction or by secreted factors [10]. A variety of secreted molecules with known immunomodulatory properties including Prostaglandin E2 (PGE2), IL-10, indoleamine 2,3-dioxygenase (IDO) and Human Leukocyte Antigen-G (HLA-G), is being produced by MSCs [10]. Among these factors, HLA-G seems to exert key immunosuppressive properties. HLA-G plays crucial role in preventing the rejection of the semiallogenic fetus by the mother, and also can be used as pre-eclampsia biomarker. HLA-G is non-classical HLA class I molecule, which is located to chromosome 6 (locus p21.1-21.3) in humans. Furthermore, HLA-G is characterized by membrane bound isoforms (HLA-G1-4) and by soluble isoforms (HLA-G5-7). These isoforms can regulate various immune responses such as the inhibition of T cell and natural killer (NK) cell proliferation, as long as the expansion of CD4^+^CD25^+^FOXP3^+^ regulatory T cells [11]. The expression of HLA-G in MSCs can be modulated by Interferon-γ (IFN-γ) and IL 10, which can be induced towards allorecognition by various immune cells such as mononuclear and dendritic cells. HLA-G also can be used as a potent marker for MSCs with improved immunosuppressive functions in order to be applied in regenerative medicine and allotransplantation.

Most times, prolonging culture and expansion of MSCs are required for obtaining sufficient cell numbers in order to be used for host immune regulation. By increasing the in vitro cultivation time, this could induce epigenetic modifications and decrease telomere length, which can affect significantly the MSC’s characteristics, such as proliferation potential, mesodermal differentiation ability and immunophenotypic properties [12]. In addition, increased cultivation of MSCs may possess a high risk for microbial contamination. Moreover, BM and adipose-derived MSCs require invasive procedures for the primary cell isolation. On the other hand, WJ MSCs have at least similar characteristics with the MSCs from the above sources and exerts the same immunoregulatory properties [13]. In this way, the vitrification and storage of WJ tissue over a long time period may be used as an alternative strategy to obtain MSCs at any desired time point. Cryopreservation by vitrification relies on the use of a combination of high and low molecular weight cryoprotective agents, protecting sufficiently the extracellular matrix (ECM) and tissue resident cells [14]. Vitrification approach reduces the ice crystal formation, thus preserving better the ECM and its mechanical properties. This approach used initially in the storage of human oocytes and embryos and its use has been extended in tissue engineering applications [14]. Several reports, have shown that WJ tissue can be cryopreserved properly followed by efficient isolation and expansion of MSCs, thus decreasing significantly the cultivation period [15,16]. However, thermomechanical stress which is induced by the vitrification and thawing procedures and the long-time storage period may alter the MSC’s functional and phernotypic characteristics, including the HLA-G expression. Until now, several research groups have evaluated the immunomodulatory properties of MSCs derived from WJ tissue, BM and adipose tissue [17,18,19]. However, little is known regarding the expression of HLA-G from MSCs derived from vitrified WJ tissue.

Under this scope, the aim of this study was to evaluate the HLA-G expression in MSCs derived either by vitrified and non-vitrified WJ tissue.

## 2. Materials and Methods

### 2.1. Isolation of WJ Tissue Segments

WJ tissue segments (n = 30, l = 7 cm) were isolated from fresh human umbilical cords that were transferred to Hellenic Cord Blood Bank (HCBB). Human umbilical cords (hUCs, n = 30, l = 10 cm) were obtained from normal and caesarian deliveries, after signed informed consent by the mothers. The informed consent for this study was in accordance with the declaration of Helsinki and approved by Institution’s Bioethics Committee. The hUCs were kept in Phosphate Buffer Saline 1x (PBS 1x, Gibco, Life Technologies, Grand Island, NY, USA) supplemented with 10 U/mL Penicillin and 10 μg/mL Streptomycin (Gibco, Life Technologies, Grand Island, NY, USA) and processed immediately to WJ tissue isolation. Briefly, the umbilical vessels were discarded and the exposed WJ tissue was isolated and transferred to 15 mL polypropylene falcon tubes (BD Biosciences Bedford, Bedford, MA, USA) with PBS 1x (Gibco, Life Technologies, Grand Island, NY, USA) until further use.

### 2.2. Vitrification of WJ Tissue

Isolated WJ tissue (n = 10, l = 2 cm), were cut into segments using sterile instruments. Specifically, each WJ tissue was divided into 3 segments with an average length of 2 cm. A number of 10 samples of WJ tissue was placed into cryotubes (BD Biosciences Bedford, Bedford, MA, USA) with approximately 2 mL of precooled VS55 vitrification solution. VS55 solution was consisted of 3.10 M DMSO, 3.10 M formamide, 2.21 M 1,2-propanediol (Sigma Aldrich, St. Louis, MO, USA) in Euro-Collins solution (IndiaMART, Noida, India).

The cryotubes contained the WJ tissue samples were rapidly cooled (43 °C/min) until reached −100 °C, followed by slow cooling (3 °C/min) to −135 °C. Finally, the samples were transferred to liquid nitrogen at −196 °C. The samples were stored in this state for a time period of 1 year. The same procedure was performed in WJ tissue samples (n = 10, l = 2 cm), without the addition of any cryoprotective agent. These tissue segments were served as positive control group and will be referred as CPA-free samples. Non-vitrified fresh WJ tissue samples (n = 10, l = 2 cm) were also used and served as negative control group for this study. 

### 2.3. Thawing of WJ Tissue

After 1 year of storage in liquid nitrogen, vitrified (n = 10, l = 2 cm) and CPA-free (n = 10, l = 2 cm) WJ tissue samples were thawed. Briefly, the cryotubes were quickly transferred from −196 °C to waterbath at 37 °C. Then, each sample was transferred to 50 mL polypropylene falcon tubes (BD Biosciences Bedford, Bedford, MA, USA) with 40 mL of PBS 1x (Gibco, Life Technologies, Grand Island, NY, USA) and centrifuged at 500× *g* for 6 min. Finally, the supernatant was discarded and the WJ tissue samples were placed to 100 mm^2^ Petri dish (ThermoFisher Scientific, Waltham, MA, USA) in order to proceed to isolation of WJ-MSCs.

### 2.4. Isolation and Expansion of WJ-MSCs

WJ tissue derived either from non-vitrified (n = 10, l = 2 cm), vitrified (n = 10, l = 2 cm) and CPA-free (n = 10, l = 2 cm) samples were trimmed with the use of sterile instruments and then each sample was placed separately in 6-well plate (Costar, Corning Life, Canton, MA, USA). Finally, 1 mL of standard culture medium was added in each well, and the 6-well plates were remained in humidified atmosphere with 5% CO_2_ at 37 °C for a total time period of 18 days. When confluency observed, the cells were detached using 0.25% trypsin-EDTA solution (Gibco, Life Technologies, Grand Island, NY, USA) and transferred to 75 cm^2^ cell culture flask (Costar, Corning Life, Canton, MA, USA). The cells remained in 75 cm^2^ cell culture flask (Costar, Corning Life, Canton, MA, USA) for additional 10 days, upon reaching confluency. Then, the cells were trypsinized and transferred to 175 cm^2^ cell culture flask (Costar, Corning Life, Canton, MA, USA). The same procedure was performed until the cells reached passage (P) 8. The standard culture medium used in this study, consisted of α-Μinimum Essentials Medium (α-ΜΕΜ, Gibco, Life Technologies, Grand Island, NY, USA) supplemented with 15% *v*/*v* fetal bovine serum (FBS, Gibco, Life Technologies, Grand Island, NY, USA) and 1% *v*/*v* penicillin (Gibco, Life Technologies, Grand Island, NY, USA) and 1% *v*/*v* streptomycin (Gibco, Life Technologies, Grand Island, NY, USA).

### 2.5. Histological Analysis of WJ Tissue

Histological analysis of non-vitrified (n = 5), vitrified (n = 5) and CPA-free (n = 5) WJ tissue samples with Hematoxylin and Eosin (H&E, Sigma-Aldrich, Darmstadt, Germany) stain, was performed. Briefly, the WJ tissue samples were fixed with 10% *v*/*v* neutral formalin buffer (Sigma-Aldrich, Darmstadt, Germany), dehydrated, paraffin embedded and sectioned at 5 μm. Then, the slides were rehydrated and stained with H&E stain. Finally, images were acquired with Leica DM LS2 (Leica, Microsystems, Wetzlar, Germany) microscope and processed with IC Capture v 2.4 software (Imaging Source, Bremen, Germany).

### 2.6. Multi-Differentiation Capacity of WJ-MSCs

The differentiation ability of WJ-MSCs towards “osteogenic”, “adipogenic” and “chondrogenic” lineages was assessed. For this purpose, WJ-MSCs P3 from non-vitrified (n = 3) and vitrified (n = 3) tissue samples were used. Specifically, WJ-MSCs at a density of 5 × 10^4^ cells were plated in each well of 6-well plates (Costar, Corning Life, Canton, MA, USA) with standard culture medium for “osteogenic” and “adipogenic” differentiation. When, the cells reached 80% of confluency, the culture medium was aspirated and briefly washes with PBS 1x (Gibco, Life Technologies, Grand Island, NY, USA) were performed. Then, PBS 1x was removed totally and the cells were subjected to differentiation.

“Osteogenic” differentiation was performed by addition of basal medium (Mesencult, StemCell Technologies, Vancouver, BC, Canada) supplemented with 15% *v*/*v* Osteogenic stimulatory supplements (StemCell technologies, Vancouver, BC, Canada), 0.01 mM dexamethasone (StemCell technologies, Vancouver, BC, Canada) and 50 ng/mL ascorbic acid (StemCell technologies, Vancouver, BC, Canada). The total time period needed for the differentiation to “osteocytes” was 25 days and then Alizarin Red-S (Sigma-Aldrich, Darmstadt, Germany) staining was performed in order to confirm the successful differentiation. WJ-MSCs were subjected to “adipogenic” differentiation by using the basal medium (Mesencult, StemCell Technologies, Vancouver, BC, Canada) supplemented with 10% *v*/*v* of adipogenic stimulatory supplements (StemCell Technologies, Vancouver, BC, Canada). After 25 days of culture, Oil Red-O (Sigma-Aldrich, Darmstadt, Germany) staining was performed.

“Chondrogenic” differentiation was conducted in 3D spheroid cultures, by transferring WJ-MSCs at a density of 35 × 10^4^ cells in 15 mL polypropylene falcon tubes (BD Biosciences Bedford, USA). “Chondrogenic” differentiation medium consisted of high glucose D-MEM (Sigma-Aldrich, Darmstadt, Germany) supplemented with 0.01mM dexamethasone (StemCell technologies, Vancouver, BC, Canada), 40 μg/mL ascorbic acid-2 phosphate (StemCell Technologies, Vancouver, BC, Canada), 10 ng/mL transforming growth factor-β1 (TGF-β1, Sigma-Aldrich, Darmstadt, Germany), and 100 μL of insulin-transferin selenium liquid medium 100x (ITS 100x, StemCell technologies, Vancouver, BC, Canada). After 30 days of culture, the pellets were fixed with 10% *v*/*v* neutral formalin buffer (Sigma-Aldrich, Darmstadt, Germany), dehydrated, paraffin embedded and sectioned at 5 μm. Alcian blue (Sigma-Aldrich, Darmstadt, Germany) was performed in each sample for the determination of “chondrogenic” differentiation. Images were acquired with Leica DM LS2 (Leica, Microsystems, Wetzlar, Germany) microscope and processed with IC Capture v 2.4 software (Imaging Source, Bremen, Germany).

### 2.7. Colony-Forming Unit-Fibroblast (CFU-F) Assay in WJ-MSCs

WJ-MSCs derived from non-vitrified (n = 3) and vitrified (n = 3) tissue samples were seeded at a density of 500 cells/well on 6-well plates (Costar, Corning Life, Canton, MA, USA), followed by addition of 1 mL of standard culture medium. The cultures remained in a humidified atmosphere with 5% CO_2_ at 37 °C for 15 days. The culture medium was changed twice a week. After 15 days of cultivation, WJ-MSCs were fixed with 10% *v*/*v* neutral formalin buffer (Sigma-Aldrich, Darmstadt, Germany) for 5 min. Finally, Giemsa (Sigma-Aldrich, Darmstadt, Germany) staining and manual counting of the colonies by two independent observers were performed. Images were acquired with Leica DM LS2 (Leica, Microsystems, Wetzlar, Germany) microscope and processed with IC Capture v 2.2 software (Imaging Source, Bremen, Germany).

### 2.8. Cell Doubling Time, Population Doubling and Cell Viability Estimation

Total cell number, cell doubling time (CDT), population doubling (PD) and cell viability was measured after each passage of WJ-MSCs until reached passage 8. Initially, WJ-MSCs at a density of 2 × 10^5^ cells were placed in 75 cm^2^ cell culture flasks (Costar, Corning Life, Canton, MA, USA).

The CDT was estimated based on the following equation

$$\mathrm{CDT}=\frac{\log_{10}\left(N/N0\right)}{\log_{10}\left(2\right)}\;x\;(T)$$

The determination of PD rate was performed according to the equation

$$\mathrm{PD}=\frac{\log_{10}\left(N/N0\right)}{\log_{10}\left(2\right)}$$

where *N* was the number of cells at the end of the culture, *N*0 was the number of seeded cells and *T* was the culture duration in hours. 

The viability of WJ-MSCs was performed using the Trypan blue (Sigma Aldrich, St. Louis, MO, USA). Total cell number was microscopically counted in Neubauer slide (Celeromics, Valencia, Spain). The cell viability and total cell number counting were performed by two independent observers. In addition, WJ-MSCs derived only from non-vitrified (n = 5) and vitrified (n = 5) tissue samples were used for the above measurements.

### 2.9. Flow Cytometric Analysis

WJ-MSCs at passage 3 derived from vitrified (n = 3) and non-vitrified (n = 3) tissue samples, were analyzed with flow cytometry for the expression of specific CDs. WJ-MSCs were tested for CD90 (Thy-1), CD105 (endoglin), CD73 (ecto-5’-nucleotidase), CD29 (integrin subunit), CD19 (pan-B-cell marker), CD31 (pan-endothelial cell marker), CD45 (pan-hematopoietic cell marker), CD34 (hematopoietic stem cell marker), CD14 (TLR-4 co-receptor), CD3 (T-cell co-receptor), CD19 (B-Cell marker), HLA-DR (HLA class II antigen) and HLA-ABC (HLA class I antigen). In addition, anti-CD90, HLA-ABC, CD29, CD19, CD31 and CD45 were conjugated with fluorescein isothiocyanate and anti-CD105, CD73, CD44, CD3, CD34, CD14 and HLA-DR were conjugated with phycoerythrin. All monoclonal antibodies used for this assay, were purchased from Immunotech (Immunotech, Beckman Coulter, Marseille, France). The flow cytometric analysis was performed in Cytomics FC 500 (Beckman Coulter, Marseille, France) coupled with the CXP Analysis software (Beckman Coulter, Marseille, France).

### 2.10. Evaluation of HLA-G Expression

HLA-G expression was evaluated in WJ-MSCs P3 derived from non-vitrified (n = 5) and vitrified (n = 5) WJ tissue samples. Total mRNA was isolated using the TRI-reagent (Sigma-Aldrich, Darmstadt, Germany) according to manufacturer’s instructions. Reverse transcription polymerase chain reaction (RT-PCR) with Omniscript RT Kit (Qiagen, Hilden, Germany) using 800 ng of RNA was performed. Complementary DNA (cDNA) was amplified with PCR by using specific primers (Table 1). Taq PCR Master Mix (Qiagen, Hilden, Germany) was applied according to manufacturer’s instructions. PCR was performed on Eppendorf Master Cycler (Eppendorf, Hamburg, Germany) and involved the following steps: initial denaturation at 95 °C for 15, denaturation at 94 °C for 30 s, annealing at 60–61 °C for 90 s and final extension at 72 °C for 3 min. The total number of cycles used in this study was 35. Finally, the PCR products were analyzed on 1% *w*/*v* agarose gel (Sigma-Aldrich, Darmstadt, Germany). 

### 2.11. Flow Cytometric Analysis of HLA-G

HLA-G expression in WJ-MSCs P3 was determined by flow cytometric analysis. Briefly, MSCs derived from non-vitrified (n = 3) and vitrified WJ (n = 3) tissue samples at a density of 10^4^ cells were placed in flow cytrometric tube. Initially, incubation with monoclonal antibody against human HLA-G (1:1000, Catalog MA1-10359, ThermoFisher Scientific, Waltham, MA, USA) was performed for 60 min. Then, 1 mL of PBS 1x was added and the samples were centrifuged at 500× *g* for 6 min. The supernatant was discarded, followed by addition of secondary FITC-conjugated mouse IgG antibody (1:100, Sigma-Aldrich, Darmstadt, Germany). The samples were incubated for 30 min at room temperature and then centrifugated at 500× *g* for 6 min. Finally, the samples were analyzed with Cytomics FC 500 (Beckman Coulter, Marseille, France) coupled with the CXP Analysis software (Beckman Coulter, Marseille, France).

### 2.12. Immunohistochemistry for HLA-G Determination

Immunohistochemistry against HLA-G was applied in non-vitrified (n = 5) and vitrified (n = 5) WJ tissue samples. Vitrified WJ tissue samples were thawed and remained with standard culture medium for 48 h in humidified atmosphere at 37 °C prior to the performance of the immunohistochemistry assay. The EnVision FLEX Mini kit, high pH (Agilent Technologies, Santa Clara, CA, USA) was used according to manufacturer’s instructions. Briefly, the tissues were cryoembedded and sectioned at 10 μm. Initially, endogenous peroxidase blocking, followed by addition of anti-HLA-G (1:1000, Catalog MA1-10359, ThermoFisher Scientific, Waltham, MA, USA), was performed. Briefly washes were performed, followed by addition of mouse secondary IgG antibody. After, 30 mins of incubation, DAB was added. Hematoxylin (Sigma-Aldrich, Darmstadt, Germany) staining was performed. Finally, the slides were dehydrated and mounted. Images were acquired with Leica DM LS2 (Leica, Microsystems, Wetzlar, Germany) microscope and processed with IC Capture v 2.4 software (Imagingsource, Bremen, Germany).

### 2.13. Mixed Lymphocyte Reaction (MLR)

Isolation of peripheral blood mononuclear cells (PBMNCs) was performed from two volunteers after signed informed consent. The informed consent was in accordance with the declaration of Helsinki and has been approved by the bioethics committee of BRFAA. The MLR was performed according to a previous published protocol [20]. After blood sampling, gradient centrifugation was performed with the use of Ficoll Solution (Sigma-Aldrich, Darmstadt, Germany) at 500× *g* for 30 min. Then, the PBMNCs from the first volunteer (stimulator cells) were treated with 25 μg/mL mitomycin (Sigma-Aldrich, Darmstadt, Germany) for 30 min at 37 °C. The PBMNCs from the second volunteer (responder cells) were used without any treatment for the MLR assay. Total cell number were estimated by two different observers using Trypan blue (Sigma-Aldrich, Darmstadt, Germany). WJ-MSCs P3 obtained from non-vitrified (n = 10) and vitrified (n = 10), were plated at a density of 1 × 10^4^ cells on U-bottom 96-well plates (Costar, Corning Life, Canton, MA, USA) with 200 μL of standard culture medium. Equal number of responder and stimulator PBMNCs were added to each well of 96 well plate (Costar, Corning Life, Canton, MA). Cultivation of 96 well plates was performed for 5 days at 37 °C in 5% CO_2_. Finally, the responder PBMNCs were counted with MTT cell growth assay kit (Sigma-Aldrich, Darmstadt, Germany). 

Specifically, MLR assay involved the following interactions between cellular populations. Stimulator cells (n = 10) without the addition of any other cell population, responder cells (n = 10) without the addition of any other cell population, interaction between stimulator (n = 10) and responder (n = 10) cells which will be referred as MLR, MLR with MSCS derived either from non-vitrified (n = 10) or vitrified (n = 10) WJ tissue samples, stimulator cells (n = 10) with MSCs from both experimental procedures (n = 20) and responder cells (n = 10) with MSCs from both experimental procedures (n = 10).

### 2.14. Indirect Immunofluorescence for HLA-G Determination

Indirect immunofluorescence against HLA-G in WJ-MSCs obtained from non-vitrified (n = 5) and vitrified (n = 5) WJ tissue samples was performed. An average of 1 × 10^4^ WJ-MSCs were seeded on culture slides (Sigma-Aldrich, Darmstadt, Germany), followed by addition of 1 mL of standard culture medium. After 10 days, the culture slides were microscopically checked and when confluency observed, the indirect immunofluorescence was performed. For this purpose, the WJ-MSCs were fixed for 10 min with 10% *v*/*v* neutral formalin buffer (Sigma-Aldrich, Darmstadt, Germany). Antigen retrieval and blocking of cells was applied in all samples, followed by addition of monoclonal antibody against human HLA-G (1:1000, Catalog MA1-10359, ThermoFisher Scientific, Waltham, MA, USA). Secondary FITC-conjugated mouse IgG antibody (1:100, Sigma-Aldrich, Darmstadt, Germany) was added. Cell nuclei became evident with DAPI staining (ThermoFisher Scientific, Waltham, MA, USA). Finally, the slides were glycerol mounted and observed under fluorescent microscope. Images were acquired with LEICA SP5 II microscope equipped with LAS Suite v2 software (Leica, Microsystems, Wetzlar, Germany).

### 2.15. Statistical Analysis

Statistical analysis was performed with GraphPad Prism v 6.01 (GraphPad Software, San Diego, CA, USA). Comparison in cell, number, viability, CDT, PD and MTT cell growth assay between the experimental conditions was performed with the unpaired nonparametric Mann–Whitney U test. Statistically significant difference between group values was considered when *p* value was less than 0.05. Indicated values are mean ± standard deviation.

## 3. Results

### 3.1. Isolation of MSCs from Vitrified WJ-Tissue

**Figure 1:**
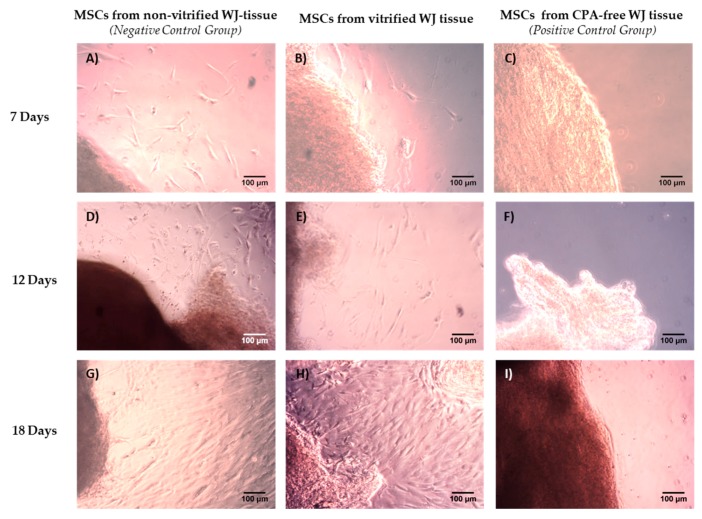
Isolation of MSCs derived from non-vitrified, vitrified and CPA-free WJ tissue. MSCs derived from fresh non-vitrified WJ-tissue (n = 10) at day 7 (**A**), 12 (**D**) and 18 (**G**). MSCs derived from vitrified WJ tissue (n = 10) at day 7 (**B**), 12 (**E**) and 18 (**H**). No MSCs were able to be isolated from CPA-free (n = 10) WJ tissue at day 7 (**C**), 12 (**F**) and 18 (**I**). Original magnification 10×, scale bars 100 μm.

MSCs were successfully isolated from fresh non-vitrified and vitrified WJ tissue samples. First evidence of cells from fresh non-vitrified and vitrified WJ tissue samples was observed at day 6 and day 7, respectively (Figure S1). Furthermore, the cells were expanded and confluency observed at day 18 (Figure 1). Then, passage of WJ-MSCs was performed. Specifically, 80% of MSCs from non-vitrified WJ-tissue samples were passaged at 75 cm^2^ cell culture flasks and 20% of MSCs were passaged at 25 cm^2^ flasks. In regards to MSCs obtained from vitrified WJ-tissue samples, the 70% were passaged at 75 cm^2^ flasks and 30% were passaged at 25 cm^2^ flasks (Figure S2).

WJ-MSCs from both experimental conditions were spindle-shaped and their morphology retained without any alteration until reaching passage 8 (Figure S3). On the other hand, no cells were isolated from CPA-free WJ tissue samples after 18 days of culture. The cultures remained for additional 10 days in a humidified atmosphere at 37 °C. No viable cells were obtained from CPA-free samples. Under this scope, for the next set of experiments only MSCs obtained from non-vitrified and vitrified WJ tissues were used.

### 3.2. Histological Analysis

**Figure 2:**
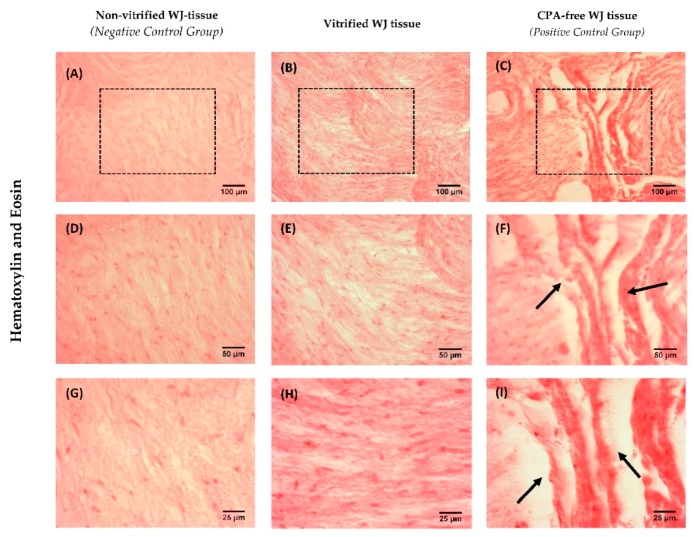
Histological analysis of WJ tissue. Non-vitrified (n = 5) WJ tissue (**A**,**D**,**G**), vitrified (n = 5) WJ tissue (**B**,**E**,**H**) and CPA-free (n = 5) WJ tissue (**C**,**F**,**I**) stained with H&E. The black boxes in 10× images (**A**–**C**) highlights the region that appeared in higher magnification of 20× and 40×. Black arrows in CPA-free WJ tissue with magnification 20× and 40× indicated the extensive damage of the tissue. Images **A**–**C**, were acquired with original magnification 10×, scale bars 100 μm. Images **D**–**F**, were acquired with original magnification 20×, scale bars 50 μm. Images **G**–**I**, were acquired with original magnification 40×, scale bars 25 μm.

Histological analysis was performed in order to determine the effect of low temperatures in WJ-tissue ultrastructure. Non-vitrified and vitrified WJ tissues were characterized by a dense gelatinous extracellular matrix, rich in resident cellular populations (Figure 2). Moreover, vitrified WJ-tissue appeared to be preserved properly with the current vitrification protocol; thus, no damage occurred in the resident cells. On the other hand, CPA-free WJ-tissue was characterized by extensive damage of the ECM, due to ice crystal formation during the storage procedure (Figure 2). The extensive WJ-tissue damage that was observed in CPA-free samples, could negatively affect the viability of WJ-MSCs.

### 3.3. Characteristics and Growth Kinetics of WJ-MSCs

**Figure 3:**
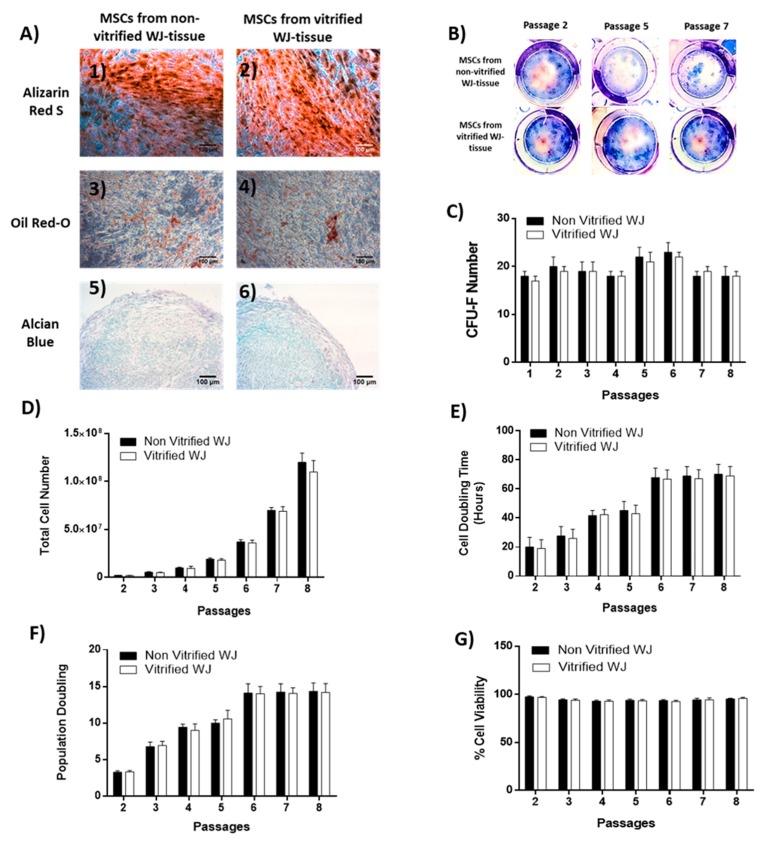
Characteristics of MSCs derived from non-vitrified and vitrified WJ tissue samples. (**A**) Differentiation potential of MSCs from non-vitrified (n = 3) WJ tissue samples towards “osteogenic (1)”, “adipogenic (3)” and “chondrogenic (5)” lineages. Differentiation potential of MSCs from vitrified (n = 3) WJ tissue samples towards “osteogenic (2)”, “adipogenic (4)” and “chondrogenic (6)” lineages. Original magnification 10×, scale bars 100 μm (**B**) CFU-F assay of WJ-MSCS obtained from non-vitrified (n = 3) and vitrified (n = 3) WJ tissue samples. (**C**) Counting of CFUs. Total cell number (**D**), CDT (**E**), PD (**F**), % cell viability (**G**) of MSCs until reaching passage 8 from non-vitrified (n = 3) and vitrified (n = 3) WJ tissue samples.

WJ-MSCs from both experimental conditions exhibited multipotent differentiation potential towards “osteogenic”, “adipogenic” and “chondrogenic” lineages as has been confirmed by Alizarin Red S, Oil red O and Alcian blue staining, respectively (Figure 3). Specifically, under “osteogenic” differentiation conditions, WJ-MSCs derived either from non-vitrified and vitrified WJ tissue samples were characterized by calcium deposits, which were visible by Alizarin Red S staining (Figure 3A). In addition, differentiated WJ-MSCs towards “adipogenic” lineages characterized by intracellular lipid droplets, which were stained positive with Oil Red-O staining (Figure 3A). Furthermore, WJ-MSCs differentiated successfully to chondrocytes and stained positive with Alcian blue staining (Figure 3A).

A CFU-F assay was performed in order to determine the clonogenic potential of WJ-MSCs. Specifically, MSCs derived from non-vitrified WJ tissue samples presented a higher number of CFU-F when compared to MSCs derived from vitrified tissue, but this increase was not statistically significant (Figure 3B,C). The higher number of CFU-F of WJ MSCs from both experimental conditions was at passage 6 (23 ± 2 and 22 ± 2 CFUs).

In addition, total cell number, CDT, PD and cell viability were calculated in order to determine the characteristics of WJ-MSCs. The total cell number of WJ-MSCs when reaching passage 8 surpassed the 1.1 × 10^8^ cells (Figure 3D). The mean CDT of MSCs derived from non-vitrified and vitrified WJ tissue samples ranged between 20 ± 7 to 70 ± 7 and 19 ± 6 to 69 ± 7 h. In addition, the mean PD of MSCs from the first experimental condition ranged between 3 ± 1 to 14 ± 1 and from the second experimental condition ranged between 3 ± 1 to 14 ± 2. The mean cell viability of MSCs derived from both experimental conditions until reaching passage 8 ranged between 94 ± 1% to 95 ± 1% and 95 ± 1% to 96 ± 1%.

### 3.4. Immunophenotypic Analysis of WJ MSCs with Flow Cytometer

WJ-MSCs from both experimental conditions expressed positively CD73, CD90, CD105, HLA-ABC, CD29 and CD44 (Table 2). In addition, WJ-MSCs derived either from non-vitrified or vitrified WJ tissue samples were characterized by negative expression of CD3, CD19, CD31, HLA-DR, CD34 and CD45 (Table 2). There were no statistically significant differences in surface markers between WJ-MSCs from both experimental conditions. A detailed list of surface marker expression is shown in Table 2.

### 3.5. Immunohistochemistry of HLA-G Expression

**Figure 4:**
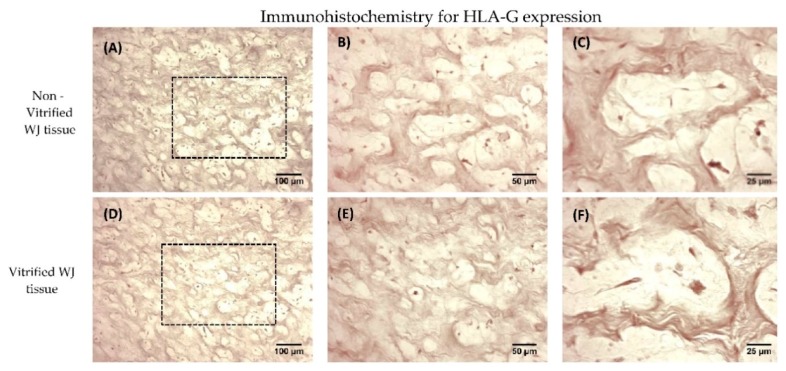
HLA-G expression in WJ tissue. Immunohistochemistry results regarding the HLA-G expression in non-vitrified (n = 5) WJ tissue (**A**–**C**). Immunohistochemistry results regarding the HLA-G expression in vitrified (n = 5) WJ tissue (**D**–**F**). The black boxes in 10× images (**A**,**D**) highlights the region that was magnified in images **B**,**C**,**E**,**F**. Images **A**,**B** were acquired with original magnification 10×, scale bars 100 μm. Images **B**,**E** were acquired with original magnification 20×, scale bars 50 μm. Images **C**,**F** were acquired with original magnification 40×, scale bars 25 μm.

Non-vitrified and vitrified WJ tissues successfully expressed the HLA-G, as indicated by immunohistochemistry results. Specifically, vitrified WJ tissue was characterized by the expression of HLA-G, even after 1 year of storage (Figure 4). In addition, non-vitrified and vitrified WJ tissues stained positive for HLA-G in a similar way as the positive control group (Figure S5).

### 3.6. HLA-G Expression in WJ-MSCs

**Figure 5:**
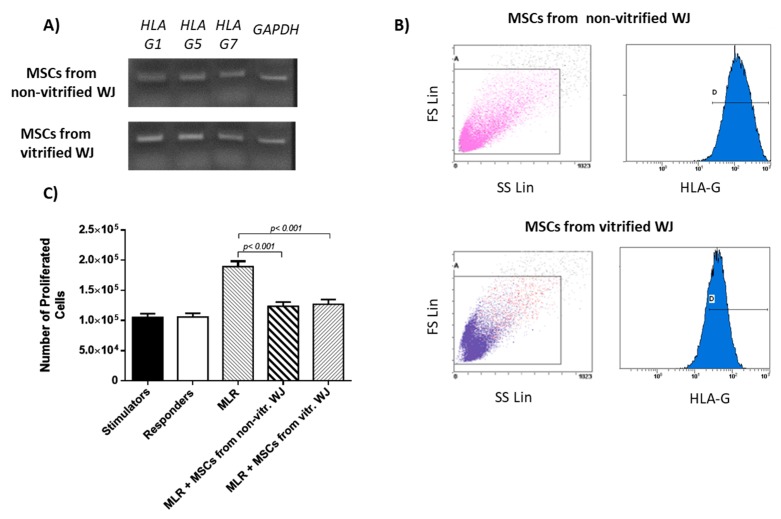
HLA-G expression by WJ-MSCs. (**A**) HLA-G expression of MSCs derived from non-vitrified (n = 5) and vitrified (n = 5) WJ tissue; (**B**) Characterization of HLA-G expression in MSCs derived from non-vitrified (n = 3) and vitrified (n = 3) WJ tissue, by flow cytometric analysis. (**C**) Mixed lymphocyte reaction using WJ-MSCs from both experimental conditions. Statistically significant differences were observed between MLR and MLR coupled with MSCs from non-vitrified WJ tissue (*p* < 0.001) and MSCs from vitrified WJ tissue (*p* < 0.001).

WJ-MSCs from both experimental conditions were characterized by the expression of *HLA-G*. Specifically, MSCs at passage 3 derived from non-vitrified and vitrified WJ tissue samples successfully expressed the intracellular isoform, *HLA-G1* and the soluble isoforms *HLA-G5* and *HLA-G7* (Figure 5A). Flow cytometric analysis indicated that 96 ± 1% of MSCs obtained from non-vitrified WJ tissue and 95 ± 2% of MSCs from vitrified WJ tissue, expressed the HLA-G (Figure 5B and Table S1).

MLR results showed that WJ-MSCs from both experimental conditions successfully suppressed the proliferation of PBMNCs as indicated by the mean number of proliferated cells (12 × 10^4^ in both experimental conditions). On the other hand, responder cells presented high proliferation (19 × 10^4^ cells) without the addition of WJ-MSCs (Table S2 and Figure S4). Statistically significant differences between MLR and MLR performed with MSCs derived either from non-vitrified (*p* <0.001) of vitrified (*p* <0.001) WJ tissue samples, were observed. No statistically significant differences were observed between MSCs derived from non-vitrified and vitrified WJ-tissue samples.

### 3.7. Indirect Immunofluorescence of HLA-G in WJ-MSCs

**Figure 6:**
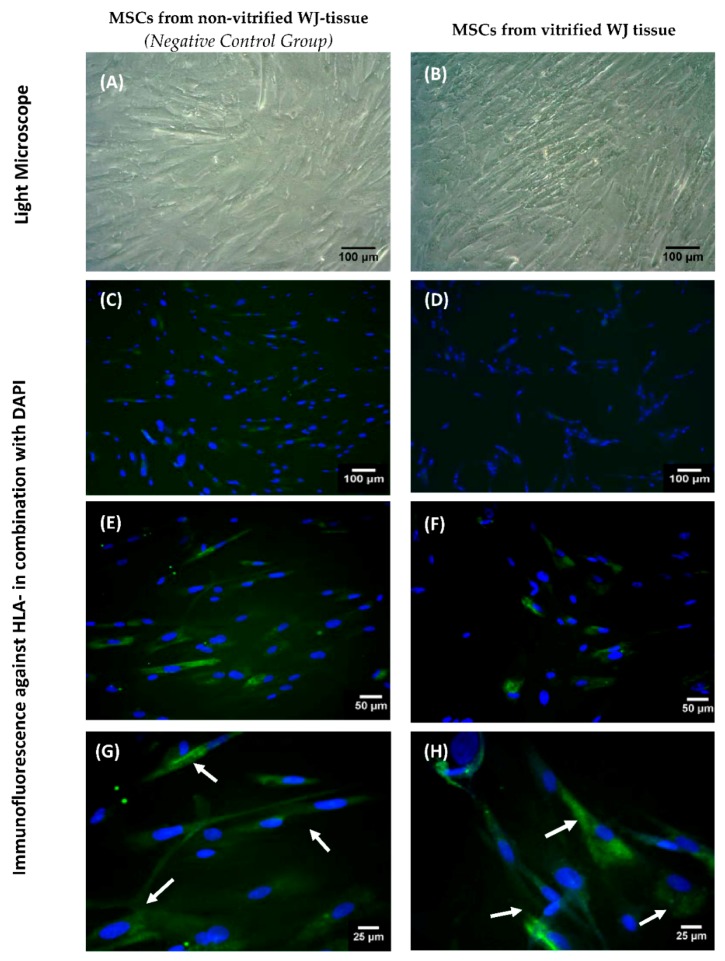
HLA-G expression in WJ-MSCs. WJ-MSCs obtained from non-vitrified (n = 5) and vitrified (n = 5) WJ tissue with light microscopy (**A**,**B**). Indirect immunofluorescence against HLA-G in combination with DAPI in MSCs derived from non-vitrified (**C**,**E**,**G**) and vitrified (**D**,**F**,**H**) WJ tissue samples. Images **A**,**B** were acquired with original magnification 10×, scale bars 100 μm. Images **C**,**D** were acquired with original magnification 10×, scale bars 100 μm. Images **E**,**F** were acquired with original magnification 20×, scale bars 50 μm. Images **G**,**H** were acquired with original magnification 40×, scale bars 25 μm.

Indirect immunofluorescence confirmed the successful expression of HLA-G by MSCs derived either from non-vitrified and vitrified WJ-tissue samples (Figure 6 and Figure S6). No alteration in staining signal was observed between MSCs obtained from both experimental conditions.

## 4. Discussion

The therapeutic applications of human MSCs towards serious life-threating disorders have been highlighted by several reports [7,8,9]. MSCs are well known for their immunomodulatory properties, which could make them ideal candidates for personalized medicine. HLA-G seems to a play crucial role in the immunosuppression process. MSCs derived from extraembryonic tissues may be characterized by higher expression of HLA-G than MSCs from other sources [10].

Under this scope, the WJ MSCs could possibly be used in several therapeutic strategies such as administration of GVHD and autoimmune disorders. In most of the times, prolonging cell culture and expansion is needed in order to obtain sufficient number of cells, which could affect significantly the MSCs properties. A possible solution to address this problem, could be the cryopreservation by vitrification of the entire WJ tissue and isolation of MSCs at any desired time point.

The aim of this study was to evaluate the HLA-G expression in MSCs derived from vitrified WJ tissue after long-term storage at −196 °C. Initially, WJ tissues were vitrified and stored for a time period of 1 year at −196 °C. Then, the tissues were thawed and MSCs were isolated. Non-vitrified and CPA-free WJ tissues stored in liquid nitrogen, were used as control groups for this study. MSCs were successfully isolated from vitrified WJ tissue and confluency observed after 18 days, in a similar way as the MSCs derived from non-vitrified WJ tissues. On the other hand, no cells were obtained from CPA-free samples. Moreover, MSCs from non-vitrified and vitrified WJ tissues characterized by the same morphology, while total cell number, CDT, PD and cell viability did not present any statistically significant difference. Furthermore, isolated MSCs from both experimental procedures were successfully passaged and reached passage 8. 

MSCs isolated from non-vitrified and vitrified WJ tissue samples were differentiated successfully to “osteocytes”, “adipocytes”, and “chondrocytes”. Furthermore, MSCs formed CFUs, were positive for CD73, CD90, CD105 and negative for CD34, CD45, HLA-DR as indicated by the ISCT [6]. Same results have been observed in several studies, elucidating in this way the efficient storage of WJ tissue over a long time period [15,16]. Moreover, histological analysis revealed that non-vitrified and vitrified WJ tissues were characterized by a dense ECM with well-preserved MSCs, while CPA-free samples exhibited extensive ECM damage. As a consequence to this, the tissue’s resident cells were not preserved properly, were damaged and no cells were isolated from CPA-free samples. This phenomenon could be explained by ice crystal formation during storage and thawing procedure of CPA-free samples [14]. On the other hand, the use of a proper combination of cryoprotective agents (low and high molecular weight) in vitrification method, resulted in the preservation of the tissue’s ECM and resident cells [14].

Once the proper storage of WJ-tissue at low temperatures was established, the expression of HLA-G was evaluated. HLA-G is expressed primarily in trophoblast and other extraembryonic tissues such as the umbilical cord. Moreover, HLA-G is elevated during pregnancy, thus maintaining in this way the immunosuppressive state towards the fetus. Moreover, the elevation of HLA-G expression is relevant with the successful implantation of trophoblast [21,22]. WJ tissue contains MSCs, which are part of the umbilical cord, thus expressing the HLA-G. Immunohistochemistry results showed the positive expression of HLA-G in WJ tissue from both experimental conditions. In order to evaluate thoroughly that MSCs from both experimental procedures expressed the HLA-G, gene expression analysis was performed.

MSCs derived either from non-vitrified or vitrified WJ-tissue successfully expressed HLA-G1, G5 and G7 isoforms. These results were in accordance with the study of Ding et al. where similar expression levels of HLA-G in MSCs derived from human umbilical cord were observed [20]. HLA-G1 is the membrane bound isoform, which is responsible for prevention of dendritic cell maturation [20]. In addition, HLA-G5 and G7 are the secreted isoforms that are implicated in immune tolerance and allograft acceptance [20]. The ability of MSCs to express both membrane-bound and soluble secreted HLA-G isoforms is very important, making them potential cell populations for treatment of several serious diseases where immune regulation is needed. Moreover, flow cytometric analysis for HLA-G showed that MSCs from both experimental procedures expressed the HLA-G at over 95%, further confirming the initial results from gene expression analysis.

The ability of MSCs to suppress immune cells was checked by an MLR assay. MSCs isolated from non-vitrified and vitrified WJ tissue samples achieved immunosuppression by decreasing the number of responder cells. On the other hand, an increase in the number of responder cells was observed when they interacted with stimulator cells without the presence of MSCs. IFN-γ, which is produced by PMNCs, is responsible for the activation of MSCs. In response to high levels of IFN-γ, MSCs express Intracellular Adhesion Molecule-1 (ICAM-1) and various immunosuppressive factors such as IDO, HLA-G and IL-10. As a result, different immune reactions can be performed either by activation of Th1 or Th2 cells. Furthermore, MSCs can efficiently modulate the immune response by activating T regulatory cells. In this study, where allorecognition was performed, MSCs were capable of suppressing the immune reaction by decreasing the number of responder PBMNCs. Finally, indirect immunofluorescence against HLA-G was performed. These results showed the positive expression of HLA-G in MSCs derived either from non-vitrified or vitrified WJ tissues.

The immunological properties of MSCs presented in the current study seemed to be consistent with previous published studies, where MSCs with different extraembryonic origins were evaluated [20,23,24,25]. In addition, several reports have focused on the evaluation of immunomodulatory properties of MSCs derived from fresh WJ tissue [20,24,25]. In our study, an initial attempt to evaluate the HLA-G expression in MSCs obtained from vitrified WJ tissue was performed. It is widely known that HLA-G is an immunomodulatory molecule which can interact with tyrosine-based immunoreceptors such as Ig-like transcript 2 (ILT2) and 4 (ILT4) and killer Ig-like receptor (KIR) 2DL4/CD158d [26,27,28]. Through this interaction, recruitment of Src homology 2 domain-containing tyrosine phosphatase 1 (SHP-1) and 2 (SHP-2), followed by inactivation of Protein Kinase B (PKB) signaling pathway, resulted in cell cycle inactivation [26,27,28,29]. In addition, HLA-G can induce T and B lymphocyte apoptosis and the activation of CD4^+^CD25^+^FoxP3^+^ regulatory T cells [18]. Due to these immunomodulatory properties, MSCs are an ideal cell population for the administration of GVHD and autoimmune disorders. GVHD and autoimmune disorders are characterized by an extensive immune reaction, where dendritic, T and B cells play crucial roles. As a first line treatment of those patients, is the use of corticosteroids. However, there are patients who develop severe or steroid-refractory GVHD or cannot respond properly to corticosteroid treatment [30,31,32]. A treatment option might be the infusion of related or unrelated MSCs. Due to their immunomodulatory properties, MSCs can induce tolerance or immune suppression to the patients, avoiding in this way the morbidity and mortality which can be caused. Future experiments will involve the use of MSCs derived from vitrified WJ tissue in animal models with occurred GVHD or autoimmune disorders [30,31,32]. In addition, the expression of HLA-G can be evaluated between MSCs from different sources such as bone marrow and adipose tissue in order to thoroughly assess their immunomodulatory properties.

## 5. Conclusions

MSCs derived from vitrified WJ tissue can efficiently retain their HLA-G expression. Cryopreservation by vitrification can be used for the proper storage of WJ tissue over a long-time period. MSCs can be isolated from vitrified WJ tissues and expanded successfully under GMP conditions, thus yielding a great number of cells that could be used in personalized medicine approaches.

## Figures and Tables

**Table 1:** Primer Set used in RT-PCR.

Gene	Forward Sequence	Target Region	Reverse Sequence	Target Region	Amplicon Size
HLA-G1	AGGAGACACGGAACACCAAG	Exon 2	CCAGCAACGATACCCATGAT	Exon 5	685
HLA-G5	AACCCTCTTCCTGCTGCTCT	Exon 1	GCCTCCATCTCCCTCCTTAC	Intron 4	895
HLA-G7	AACCCTCTTCCTGCTGCTCT	Exon 1	TTACTCACTGGCCTCGCTCT	Intron 2	331
GAPDH	AAGGGCCCTGACAACTCTTT	-	CTCCCCTCTTCAAGGGGTCT	-	244

**Table 2:** Flow cytometric analysis of MSCs derived from non-vitrified (n = 3) and vitrified (n = 3) WJ-tissue samples.

Clusters of Differentiation	MSCs Derived from Non-Vitrified WJ Tissue (*% Expression*)	MSCs Derived from Vitrified WJ Tissue (*% Expression*)	*p* Value
CD73	97.3 ± 0.9	97.0 ± 0.8	0.7431
CD90	96.0 ± 0.6	96.7 ± 0.5	0.6338
CD105	96.3 ± 0.7	96.7 ± 0.5	0.5897
HLA-ABC	97.0 ± 0.1	96.4 ± 2.3	0.7369
CD29	96.0 ± 1.3	95.2 ± 1.5	0.6306
CD44	96.5 ± 0.6	95.2 ± 0.8	0.1489
CD3	1.6 ± 0.2	1.6 ± 0.1	0.9989
CD19	1.3 ± 0.1	1.3 ± 0.5	0.2663
CD31	1.4 ± 0.1	1.4 ± 0.1	0.9978
CD14	1.1 ± 0.2	1.1 ± 0.1	0.8419
HLA-DR	1.1 ± 0.1	1.1 ± 0.1	0.6033
CD-34	1.5 ± 1.1	1.6 ± 0.2	0.4501
CD45	1.4 ± 0.3	1.3 ± 0.1	0.7445

## Supplementary Materials

The following are available online at http://www.mdpi.com/2306-5354/5/4/95/s1, Figure S1: Days of first evidence of isolated MSCs from non-vitrified, vitrified and CPA-free WJ tissue samples. Figure S2: Initial passage of MSCs to cell culture flasks. Figure S3: Passages of MSCs derived from non-vitrified and vitrified WJ tissue samples from 1 to 8. Table S1: Flow cytometric analysis of HLA-G expression in WJ -MSCs. Table S2: MLR results. Figure S4: Mixed Lymphocyte reaction. Figure S5: HLA-G expression in WJ tissue. Figure S6: Indirect immunofluorescence for HLA-G expression in WJ-MSCs.
