# Supplementary material for: Evaluation of HLA-G Expression in Multipotent Mesenchymal Stromal Cells Derived from Vitrified Wharton’s Jelly Tissue
Source: Bioengineering (Basel). 2018 Nov 1;5(4):95. doi: 10.3390/bioengineering5040095 (PMC6316308; doi:10.3390/bioengineering5040095)
Supplement: Supplementary file 1 [file bioengineering-05-00095-s001.pdf]

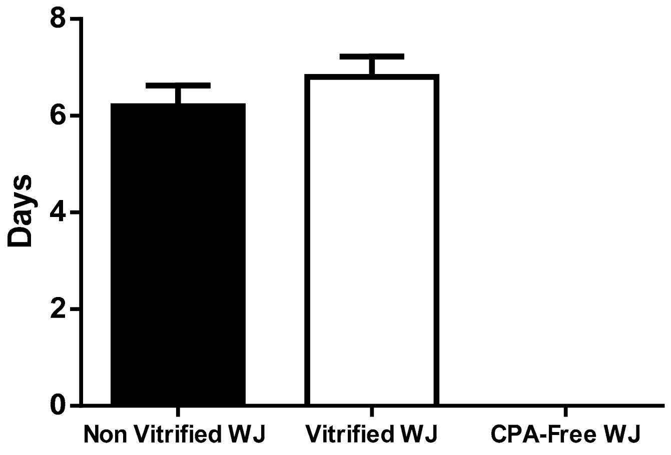


**Figure S1.** Days of first evidence of isolated MSCs from non-vitrified, vitrified and CPA-free WJ tissue samples. No MSCs were able to be isolated from CPA-free WJ tissue samples.

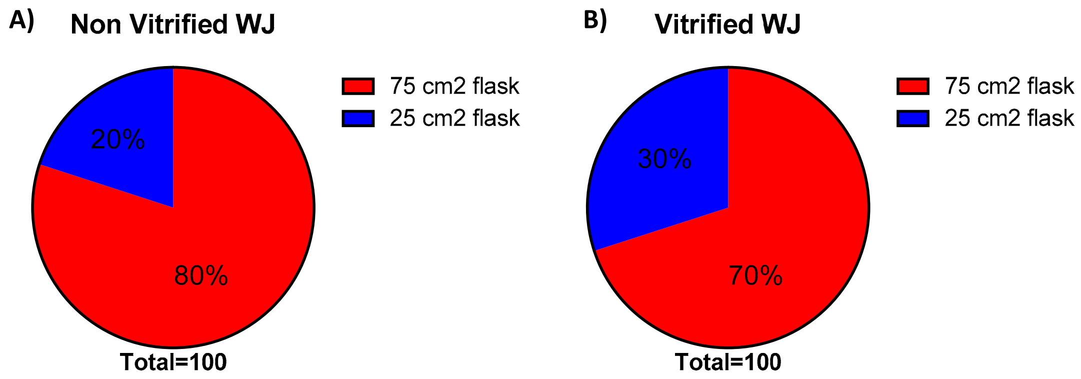


**Figure S2.** Initial passage of MSCs to cell culture flasks. A) Passage of MSCs derived from non-vitrified WJ tissue, where 80% was passaged to 75 $cm^2$ cell culture flask and the remaining 20% to 25 $cm^2$ cell culture flask. B) A) Passage of MSCs derived from vitrified WJ tissue, where 70% was passaged to 75 $cm^2$ cell culture flask and the remaining 30% to 25 $cm^2$ cell culture flask.

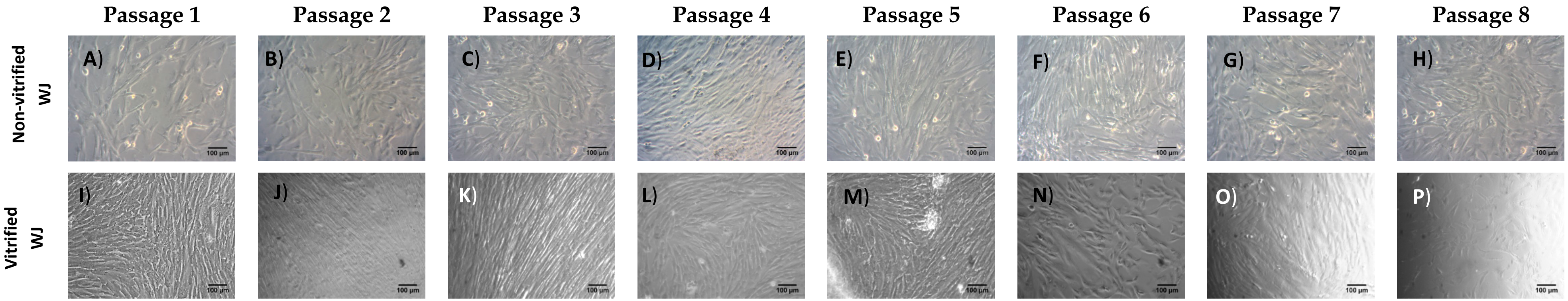


**Figure S3.** Passages of MSCs derived from non-vitrified and vitrified WJ tissue samples from 1 to 8. Passages of MSCs obtained from non-vitrified WJ tissue samples (A-H). Passages of MSCs obtained from non-vitrified WJ tissue samples (I-P). Original magnification 10x, scale bars 100 µm.

**Table S1.** Flow cytometric analysis of HLA-G expression in WJ -MSCs.

| Cluster of Differentiation | MSCs derived from non-vitrified WJ tissue *(% Expression)* | MSCs derived from vitrified WJ tissue *(% Expression)* | *p v*alue |
|---|---|---|---|
| HLA-DR | 95.6 ± 1.2 | 95.3 ± 1.6 | 0.84349 |

**Table S2.** MLR results

| | Stimulator Cells | Responder Cells | Stimulators with MSCs from non-vitrified WJ | Responders with MSCs from non-vitrified WJ | Stimulators with MSCs from vitrified WJ | Responders with MSCs from Vitrified WJ | MLR | MLR with MSCs from non-vitrified WJ | MLR with MSCs from vitrified WJ |
|---|---|---|---|---|---|---|---|---|---|
| | 98000 | 100000 | 101000 | 95000 | 101200 | 110000 | 176000 | 121000 | 117000 |
| | 110000 | 110000 | 120000 | 110000 | 120000 | 110000 | 190000 | 135000 | 142000 |
| | 102000 | 109000 | 102000 | 109000 | 102000 | 109000 | 182000 | 132000 | 125000 |
| | 120000 | 102000 | 121500 | 102000 | 110000 | 112500 | 201000 | 114000 | 129000 |
| | 101000 | 120000 | 101000 | 120000 | 115000 | 120000 | 179000 | 125000 | 131000 |
| | 106000 | 107000 | 106500 | 107000 | 106000 | 107000 | 195000 | 122000 | 117000 |
| | 103000 | 98000 | 104500 | 115000 | 99500 | 98000 | 197000 | 123000 | 124000 |
| | 102000 | 103000 | 102000 | 103000 | 120000 | 103000 | 182000 | 124000 | 127000 |
| | 106000 | 107000 | 104890 | 106500 | 106000 | 107000 | 189000 | 127000 | 122000 |
| | 105000 | 104000 | 104000 | 104000 | 112500 | 104000 | 201000 | 114000 | 135000 |
| Average | 105300 | 106000 | 106739 | 107150 | 109220 | 108050 | 189200 | 123700 | 126900 |
| Standard Deviation | 5815 | 5933 | 7215 | 6633 | 7133 | 5623 | 8693 | 6388 | 7395 |

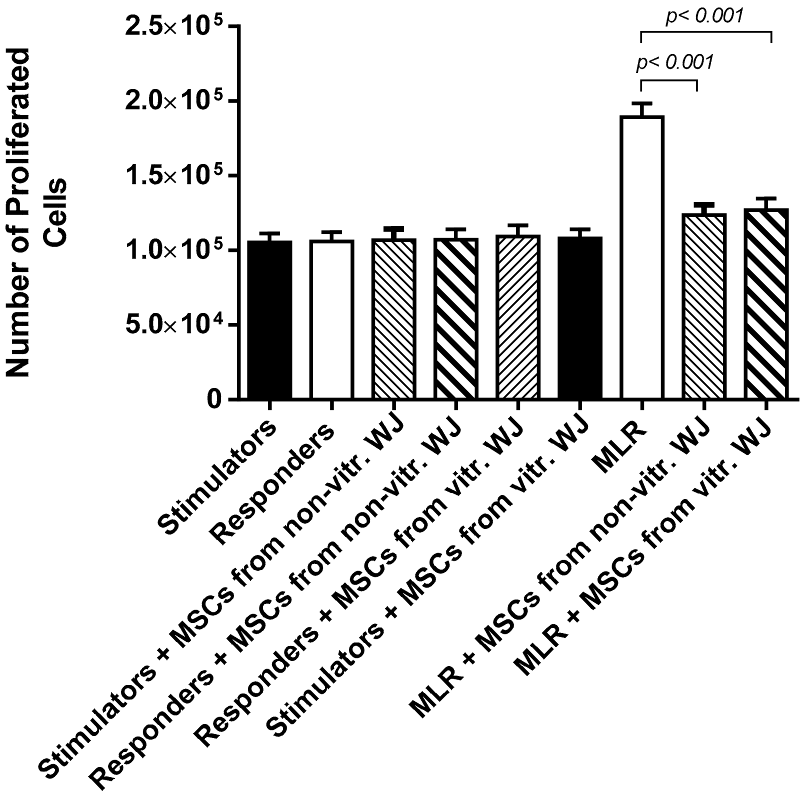


**Figure S4.** Mixed Lymphocyte reaction. Statistical significant difference was observed between MLR and MLR coupled with either MSCs from non-vitrified WJ tissue (*p<0.001*) or MSCs from vitrified WJ tissue (*p<0.001*).

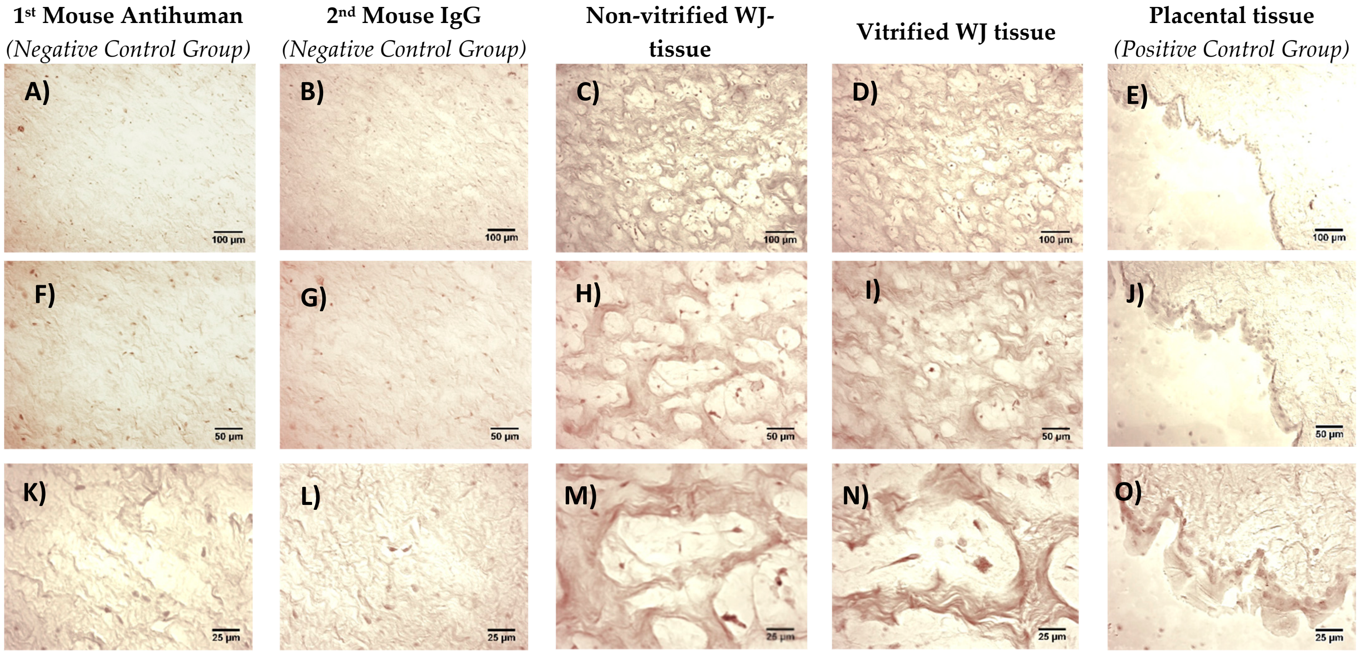


**Figure S5.** HLA-G expression in WJ tissue. 1st mouse antihuman – negative control group (A, F, K). 2nd Mouse IgG – Negative Control Group (B, G, L). Non-vitrified WJ-tissue (C, H, M). Vitrified WJ tissue (D, I, N). Placental tissue -Positive Control Group (E, J, O). Images A-E were acquired with original magnification 10x, scale bars 100 µm. Images F-J, were acquired with original magnification 20x, scale bars 50 µm. Images K-O, were acquired with original magnification 40x, scale bars 25 µm.

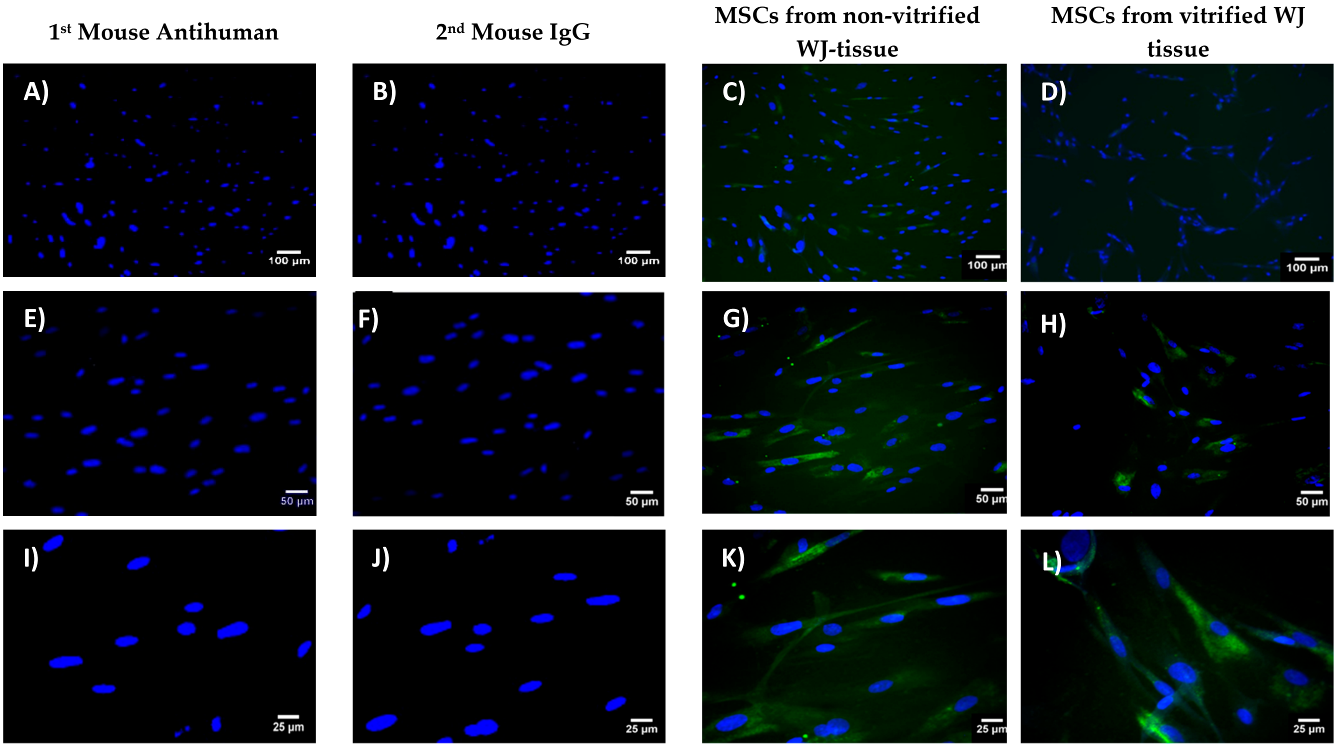


**Figure S6.** Indirect immunofluorescence for HLA-G expression in WJ-MSCs. 1st Mouse Antihuman monoclonal antibody against HLA-G (A,E,I). 2nd Mouse IgG monoclonal antibody (B, F, J). MSCs from non-vitrified WJ-tissue (C, G, K) and from vitrified WJ-tissuw (D, H, L). Images A-D were acquired with original magnifcation 10x, scale bars 100 µm. Images E- H, were acquired with original magnification 20x, scale bars 50 µm. Images I-L, were acquired wit original magnification 40x, scale bars 25 µm.
